# Association between the lactate to creatinine ratio and hospital mortality in patients with pediatric sepsis: a cohort study

**DOI:** 10.1038/s41390-025-04751-1

**Published:** 2026-01-21

**Authors:** Qiyin Cai, Xin Ye, Yongtong He, Qin Liu, Jiali Huang, Guanghui Chen, Chaofu Qin

**Affiliations:** 1 Department of Pediatric Intensive Care Units, Jiangmen Maternity and Child Health Care Hospital, Jiangmen, China; 2 Department of Neurosurgery, Affiliated Jiangmen TCM Hospital of Ji’nan University, Jiangmen, China

## Abstract

**Background:**

The relationship between lactate to creatinine ratio (LCR) and prognosis in pediatric sepsis is poorly understood. Our study aimed to investigate the association between LCR and hospital mortality in pediatric sepsis patients.

**Methods:**

A retrospective cohort study of 4379 patients with pediatric sepsis, categorized by LCR quartiles, was performed. The association between LCR and hospital mortality was investigated using univariate and multivariate logistic regression models, as well as subgroup and sensitivity analyses. Restricted cubic splines were employed to explore non-linear relationships, and a two-piece logistic proportional hazards model was constructed for both sides of the inflection point.

**Results:**

The overall hospital mortality rate for pediatric sepsis was 9.6%. Following adjustment for relevant covariates, a positive correlation between LCR and hospital mortality was observed. A nonlinear relationship between the baseline LCR and the risk of hospital mortality was identified, with LCR < 0.780 associated with a significantly higher risk. Beyond the LCR turning point of 0.780, the risk did not substantially increase.

**Conclusions:**

The study revealed a nonlinear relationship between the LCR and hospital mortality in children with sepsis. When the LCR is less than 0.780, hospital mortality rises drastically as the LCR advances.

**Impact:**

- This research delves into the non-linear correlation between the lactate to creatinine ratio (LCR) and hospital mortality in pediatric sepsis patients. It identifies a critical threshold of LCR < 0.780 linked to higher mortality rates.
- This study presents the first comprehensive analysis of LCR’s significance in pediatric sepsis, suggesting it as a potential biomarker for risk assessment and clinical decision-making.
- The implications of these findings could potentially lead to improved treatment strategies, ultimately decreasing hospital mortality in children with sepsis by optimizing lactate and creatinine management.

## Introduction

Sepsis is a dangerous systemic inflammatory response caused by microbial infection and is a serious threat to the health of children worldwide.^1,2^ It can lead to organ damage, shock, and even death, affecting ~25.2 million children each year and causing 3.3 million deaths.^3,4^ In an effort to improve the early identification and treatment of sepsis in children, the International Society of Critical Care Medicine Pediatric Sepsis Definition Task Force introduced the Phoenix Sepsis Criteria in 2024.^5^ These criteria aim to provide clear guidelines for the diagnosis of pediatric sepsis and septic shock. Additionally, researchers are actively working to identify biomarkers that may aid in early detection and prognosis to improve clinical outcomes for patients with sepsis.

Sepsis is a potentially fatal illness caused by an uncontrolled reaction to infection, frequently accompanied by high lactate and creatinine levels.^6^ Lactate, which is formed when the tissue is depleted of oxygen, is an important sign of sepsis severity, and its clearance rate can help predict patient survival.^7,8^ However, it is crucial to highlight that lactate levels can also rise owing to non-specific causes. The liver and kidneys play important roles in lactate utilization, and disorders affecting these organs might impair lactate clearance, potentially leading to incorrect measurements of tissue oxygen levels.^9,10^ Early circulatory irregularities and systemic hypoxia in sepsis can cause acute kidney damage, which is often deadly. Creatinine, an early biomarker of acute kidney injury reveals tissue damage and the severity of severe sickness during inflammatory responses.^11^ However, its sensitivity is restricted, and it exhibits delayed reactivity.^12^ Given these interdependent limitations, the serum lactate-to-creatinine ratio (LCR) emerges as a composite biomarker integrating two pathophysiological axis: lactate (tissue hypoxia/anaerobic metabolism) and creatinine (renal function). Whereas isolated lactate interpretation risks confounding by non-hypoxic factors, and creatinine alone inadequately captures dynamic perfusion changes, LCR provides multidimensional assessment of metabolic-hemodynamic status. In sepsis, the concurrent evaluation of tissue perfusion and renal function is crucial for prognosis, and this dual biomarker approach may enhance predictive accuracy.

To validate this paradigm, we hypothesized that LCR correlates with pediatric sepsis prognosis. Despite its pathophysiological rationale, the association between LCR and in-hospital mortality remains underexplored. This study aimed to assess the association between the LCR and in-hospital mortality in pediatric sepsis and to investigate the possibility of a non-linear dose-response relationship.

## Methods

### Data source

The data were derived from PLOS ONE (https://journals.plos.org/plosone/), which allows unrestricted access to the original data. We cited the PLOS ONE dataset (“Data from: Validating the performance of organ dysfunction scores in children with infection: A cohort study,” PLOS ONE, Dataset, https://doi.org/10.1371/journal.pone.0306172).^13^

### Ethical approval

The study was authorized by the Ethics Committee of the Children’s Hospital of Chongqing Medical University, China, and the data were anonymized so that the investigators could not identify individual participants during or after collection. The protocol has been registered with the Chinese Clinical Trials Registry with the identifier ChiCTR2100053198. We conducted a secondary analysis of the dataset. This study did not require an additional ethical declaration because the public policy statement connected to the dataset had previously been approved by the research ethics committee.

### Study population and setting

This retrospective cohort analysis included patients aged 4 weeks to 18 years who were admitted to the Children’s Hospital of Chongqing Medical University Pediatric Intensive Care Unit (PICU) with suspected or confirmed infection between 2015 and 2021. Patients were excluded for (1) missing outcome data, (2) Phoenix Sepsis Score (PSS) of less than two,^5,14^ and (3) stayed in the PICU for less than one day. We reported this study in accordance with the Strengthening the Reporting of Observational Studies in Epidemiology (STROBE) guidelines for observational studies.

### Data collection

On the first day of PICU admission, demographic information (age group and sex), vital signs (respiratory rate [RR], heart rate [HR], temperature, mean arterial pressure [MAP], basic disease, Glasgow coma score, and respiratory failure [PO_2_/FiO_2_ < 60 mmHg]) were obtained from the Sepsis Specialized Disease Database. Routine laboratory tests such as PaCO_2,_ PaO_2,_ FiO_2,_ platelet count, white blood cell count (WBC), procalcitonin (PCT), C-reactive protein (CRP), blood urea nitrogen (BUN), lactate, serum creatinine, bilirubin, and fibrinogen were also obtained. The LCR was computed (LCR = lactate/serum creatinine).^15,16^ Treatment information, such as mechanical ventilation (MV), vasopressor usage, extracorporeal membrane oxygenation, and renal replacement therapy (RRT) were documented. The lengths of PICU stays and hospitalization were also recorded. Each participant’s time was measured from the cohort admission date to the date of death or discharge. All laboratory values were collected during the first 24 h of PICU admission.

### Definition

This study classified pediatric sepsis as a suspected infection and PSS ≥ 2, which is made up of four organ dysfunctions: respiratory, cardiovascular, coagulation, and neurological.^5^ For children under 2 years, age-specific MAP reference values required to calculate the cardiovascular subscore were unavailable because the dataset did not provide stratification by the three pediatric age bands (4 weeks–1 month, 1–11 months, and 1–2 years). In these cases, cardiovascular dysfunction was defined using surrogate indicators—serum lactate ≥2 mmol/L or the use of vasoactive agents.^17^

### Endpoint

^The outcome was defined to be hospital mortality.^

### Statistical analysis

The Kolmogorov-Smirnov test was used to assess the normality of the variables. Normally distributed continuous data are expressed as mean ± standard deviation, while skewed continuous variables are expressed as median (interquartile range [IQR]). Categorical variables were reported using frequencies and percentages. A paired t-test or a Wilcoxon signed-rank test was used to examine differences within groups. The chi-squared or Fisher’s exact test was employed for categorical variables, the one-way analysis of variance test for normally distributed variables, and the Kruskal-Wallis H test for skewed distributions.

Univariate and multivariate logistic regression analyses were used to investigate the relationship between the LCR and hospital mortality. The LCR was evaluated as a categorical variable (four quantiles) to facilitate clinical interpretation and to reduce the influence of extreme values, given its skewed distribution. Confounding variables were selected based on their clinical relevance, preceding scientific literature, significance in univariate analysis, and any changes in effect estimates that exceeded 10%. Variance inflation factor values > 5 indicate multicollinearity.^18^ Three models were created: Model 1 accounted for age and sex. Model 2 was further adjusted for fundamental disease, respiratory failure, MAP, and PSS. Model 3 was further modified to account for WBC, PCT, CRP, BUN, bilirubin, MV, RRT, and vasopressor usage. Trend analyses were performed using multivariate logistic regression models.

Restricted cubic spline models were used to explore potential nonlinear relationships between the LCR and hospital mortality. A likelihood ratio test comparing models with linear terms only to those with both linear and cubic spline terms was used to evaluate non-linearity. Additionally, a two-piecewise linear regression model based on the smoothing curve was built to detect potential threshold effects while accounting for confounders. Less than 5% of the dataset contained missing data, which was removed listwise during the analysis. Subgroup variables were used for interaction and stratified analyses, and likelihood ratio tests were used to investigate subgroup interactions. Additionally, a sensitivity analysis was performed on patients who underwent a PSS assessment without a cardiovascular score, patients ≥2 years with complete cardiovascular data, excluding outliers and excluding patients on RRT. This analysis aimed to determine the trustworthiness of the study’s findings and the potential impact of using different models to infer correlations. The effect sizes and *P* values derived from these models were presented and compared for a comprehensive evaluation. A two-sided *P*-value < 0.005 was considered statistically significant.

For every analysis, the Free Statistics analytical platform (version 2.0, Beijing, China, http://www.clinicalscientists.cn/freestatistics) and R Statistical Software (version 4.2.2, http://www.R-project.org, The R Foundation) were used.

## Results

The study initially enrolled 5609 patients with suspected or confirmed infections; however, following screening, 4379 patients with pediatric sepsis were included in the current analysis. Exclusion criteria included 253 patients with missing outcome data, 874 patients with PSS less than two, and 103 patients who lasted less than one day in the PICU (Fig. 1). The overall prevalence of hospital mortality was 9.6%. The median PICU length of stay was 6.5 days (IQR: 3.0, 12.0), and the median hospital length of stay was 24 days (IQR: 15.0, 34.0).

**Fig. 1:**
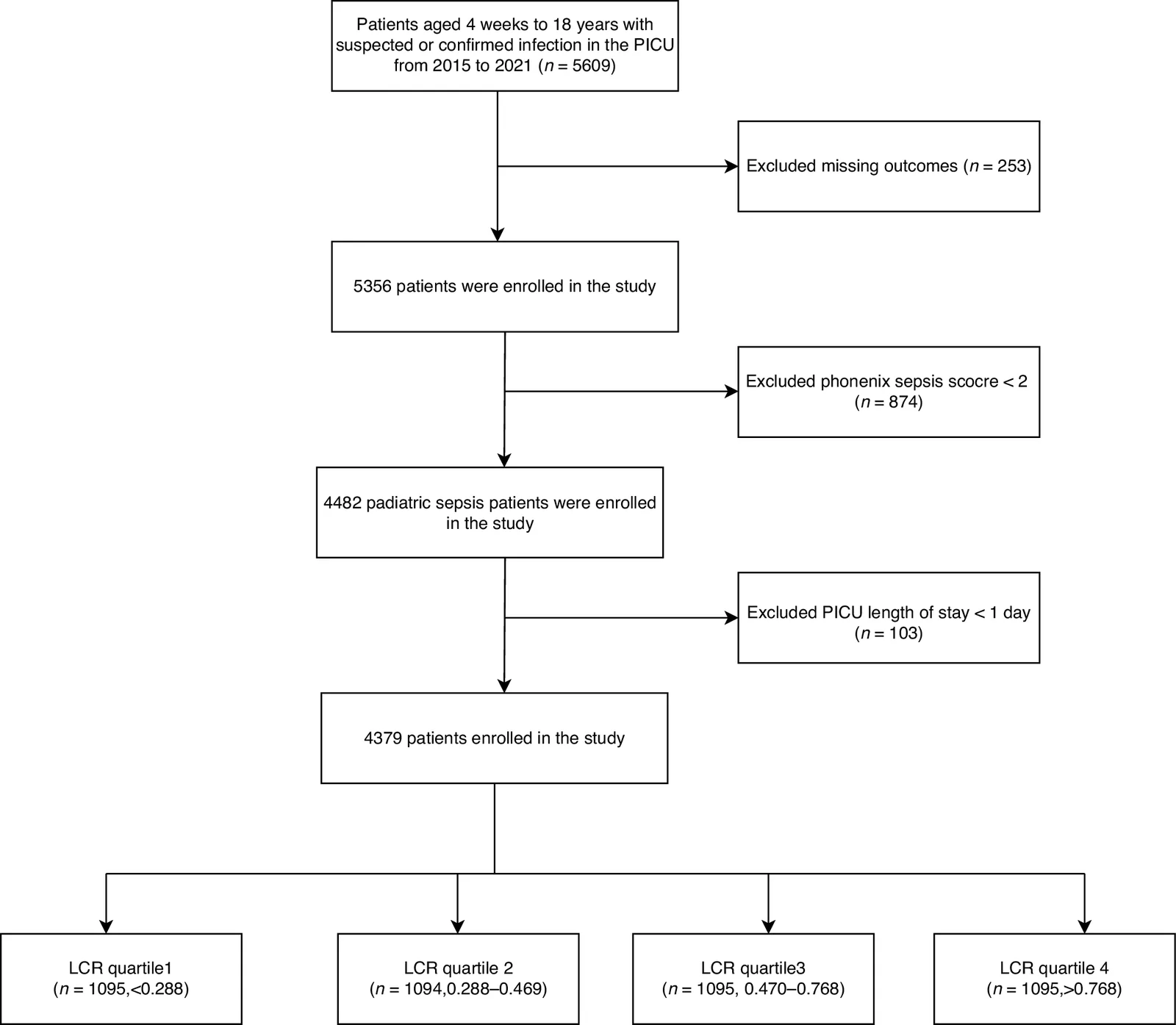
Flowchart of participant selection. Numbers of patients at each stage of inclusion and exclusion are shown.

### Baseline characteristics of study participants

The baseline clinical characteristics of the 4379 enrolled patients are summarized in Table 1. The study cohort was divided into quartiles based on the LCR levels (Q1 < 0.288, Q2 0.288– 0.469, Q3 0.470–0.768, and Q4 > 0.768). Most patients (64.7%) were in the age group of 4 weeks to 2 years, with the highest percentage noted in Q1 (65.2%). There were slightly more male patients (59.4%), with the lowest quartile of LCR having the highest percentage of male patients (61.3%). The mean MAP was similar between the LCR groups (*P* = 0.436). The mean PSS at Q4 was higher than at Q1 (3.5 ± 1.4 vs. 2.8 ± 0.9, *P* < 0.005). The median WBC in Q4 was the highest (13.1, IQR: 8.9, 19.4, *P* = 0.002), while the median PCT in Q1 was the highest (1.9, IQR: 0.3, 14.9, *P* < 0.005). Median lactate in Q1 was the lowest (10.8, IQR: 8.1, 16.2, *P* < 0.005). The mean creatinine, BUN, and bilirubin in Q1 were higher than in Q4. A quarter of the patients (25.9%) had a basic disease, and 3.2% had respiratory failure. There were also significant differences in vasopressor usage and RRT (*P* < 0.005).

**Table 1:** Baseline clinical characteristics of the study population

Variables	Total (*n* = 4379)	LCR				*P* value
		Q1 (*n* = 1095, <0.288)	Q2 (*n* = 1094,0.288–0.469)	Q3 (*n* = 1095,0.470–0.768)	Q4 (*n* = 1095, >0.768)	
Age group, *n* (%)						0.000*
4 weeks–2 years	2834 (64.7)	714 (65.2)	714 (65.3)	700 (63.9)	706 (64.5)	
2–5 years	638 (14.6)	131 (12)	165 (15.1)	159 (14.5)	183 (16.7)	
5–12 years	652 (14.9)	153 (14)	155 (14.2)	174 (15.9)	170 (15.5)	
12–18 years	255 (5.8)	97 (8.9)	60 (5.5)	62 (5.7)	36 (3.3)	
Sex, *n* (%)						0.001*
Male	2600 (59.4)	671 (61.3)	687 (62.8)	638 (58.3)	604 (55.2)	
Female	1779 (40.6)	424 (38.7)	407 (37.2)	457 (41.7)	491 (44.8)	
RR(beats/min)	46.3 ± 11.2	45.4 ± 10.8	46.0 ± 10.8	46.5 ± 11.0	47.2 ± 12.2	0.002*
HR(beats/min)	167.3 ± 23.2	164.4 ± 23.3	165.8 ± 22.9	167.1 ± 22.2	172.0 ± 23.6	0.000*
Temperature (°C)	38.3 ± 1.0	38.4 ± 0.9	38.4 ± 0.9	38.3 ± 1.0	38.3 ± 1.0	0.363
MAP (mmHg)	94.8 ± 13.8	94.7 ± 13.6	94.2 ± 13.3	95.1 ± 14.0	95.0 ± 14.5	0.436
GCS	12.3 ± 2.7	12.6 ± 2.3	12.7 ± 2.4	12.4 ± 2.7	11.5 ± 3.1	0.000*
PSS	3.0 ± 1.1	2.8 ± 0.9	2.9 ± 0.9	2.9 ± 1.0	3.5 ± 1.4	0.000*
PCO_2_ (cmH_2_O)	53.6 ± 15.9	51.0 ± 14.4	52.7 ± 14.7	54.7 ± 16.0	56.1 ± 17.9	0.000*
PO_2_/FiO_2_	221.1 (177.6, 318.3)	230.2 (188.3, 326.3)	216.9 (176.0, 304.5)	221.6 (169.1, 311.0)	220.7 (166.2, 324.8)	0.000*
WBC (×10^9^/L)	12.3 (8.5, 17.8)	12.2 (8.6, 17.3)	12.1 (8.1, 17.3)	12.0 (8.5, 17.4)	13.1 (8.9, 19.4)	0.002*
PLT (×10^9^/L)	338.0 (207.0, 489.5)	281.0 (180.5, 420.5)	341.0 (219.5, 483.0)	380.0 (239.0, 525.5)	355.0 (203.5, 525.5)	0.000*
PCT(ng/mL)	1.0 (0.2, 7.7)	1.9 (0.3, 14.9)	0.9 (0.2, 6.7)	0.7 (0.1, 4.3)	1.0 (0.2, 7.3)	0.000*
CRP(mg/L)	18.0 (5.0, 46.0)	18.0 (5.0, 46.0)	19.5 (5.0, 46.0)	17.0 (5.0, 44.0)	17.0 (5.0, 47.0)	0.471
Lactate(mg/dl)	17.1 (10.8, 28.8)	10.8 (8.1, 16.2)	12.6 (9.0, 18.0)	17.1 (13.5, 25.2)	36.0 (24.3, 63.9)	0.000*
Creatinine (mg/dl)	35.0 (25.0, 57.3)	63.3 (39.8, 109.6)	33.2 (25.0, 48.4)	29.4 (22.6, 41.1)	28.8 (22.0, 42.8)	0.000*
Bilirubin (umol/l)	8.5 (3.5, 18.8)	10.2 (4.8, 18.6)	7.8 (3.0, 15.7)	7.0 (3.0, 17.8)	9.0 (3.7, 26.1)	0.000*
BUN (umol/L)	4.8 (3.2, 7.5)	6.1 (3.9, 11.2)	4.8 (3.3, 7.1)	4.4 (2.9, 6.6)	4.5 (3.0, 6.5)	0.000*
Fibrinogen (mg/dl)	2.2 (1.5, 3.4)	2.1 (1.4, 3.2)	2.3 (1.6, 3.4)	2.3 (1.6, 3.4)	2.1 (1.4, 3.4)	0.000*
Basic disease, *n* (%)	1135 (25.9)	243 (22.2)	256 (23.4)	307 (28)	329 (30)	0.000*
Respiratory failure, *n* (%)	138 (3.2)	36 (3.3)	33 (3)	26 (2.4)	43 (3.9)	0.216
MV, *n* (%)	3917 (89.4)	985 (90)	982 (89.8)	970 (88.6)	980 (89.5)	0.734
Vasopressor usage, *n* (%)	922 (21.1)	215 (19.6)	197 (18)	211 (19.3)	299 (27.3)	0.000*
RRT, *n* (%)	258 (5.9)	97 (8.9)	40 (3.7)	40 (3.7)	81 (7.4)	0.000*
Hospital mortality, *n* (%)	420 (9.6)	55 (5)	67 (6.1)	93 (8.5)	205 (18.7)	0.000*
Hospital length of stay(days)	24.0 (15.0, 34.0)	21.0 (13.0, 31.8)	21.0 (13.0, 34.0)	19.0 (9.0, 32.0)	24.0 (15.0, 34.0)	0.000*
PICU length of stay(days)	6.5 (3.0, 12.0)	7.0 (4.0, 14.0)	7.0 (3.0, 14.0)	6.5 (3.0, 12.0)	7.0 (4.0, 14.0)	0.000*

Data presented are mean ± SD, median (Q1–Q3), or *N* (%).

*P < 0.0005.

*LCR* lactate to creatinine ratio, *RR* respiratory rate, *HR* heart rate, *MAP* mean arterial pressure, *GCS* glasgow coma score, *PSS* Phoenix Sepsis Score, *PCO*_2_ partial pressure of carbon dioxide, *PO*_2_ partial pressure of oxygen, *FiO*_2_ fractional inspired oxygen, *WBC* white cell count, *PLT* platelet count, *PCT* procalcitonin, *CRP* C-reactive protein, *BUN* blood urea nitrogen, *MV* mechanical ventilation, *RRT* renal replacement therapy, *PICU* Pediatric Intensive Care Unit.

### Results of logistic regression analysis

Univariate regression analysis (Supplementary Table S1) was performed to identify factors related to hospital mortality. The study found that age, RR, HR, temperature, MAP, PSS, PaCO2, WBC, PCT, CRP, bilirubin, BUN, basic disease, respiratory failure, MV, vasopressor usage and RRT were all substantially linked with hospital mortality (all *P* < 0.005).

Multivariate logistic regression analysis was performed using four different models, with the odds ratios (OR) and 95% confidence intervals (95% CI) presented in Table 2. The models include a crude model, unadjusted; model 1, adjusted for age and sex. model 2, adjusted for model 1 plus basic disease, respiratory failure, MAP, and PSS; and model 3, adjusted for model 2 plus WBC, PCT, CRP, BUN, bilirubin, MV, RRT, and vasopressor usage. When the LCR level was considered a continuous variable, it was significantly associated with hospital mortality in the crude model, with an OR of 2.26 and a 95% CI of 1.97–2.58 (*P* < 0.005). This association remained significant in the fully adjusted model 3, with an OR of 1.48 and a 95% CI of 1.25–1.75 (*P* < 0.005). When LCR was categorized, compared with the lower group, the fully adjusted model 3 ORs were 1.41 (95% CI: 0.92– 2.16, *P* = 0.117), 2.00 (95% CI: 1.32–3.04, *P* < 0.005), and 2.94 (95% CI: 1.94–4.45, *P* < 0.005) in the Q2, Q3, and Q4, respectively.

**Table 2:** Multivariate logistic regression analysis of lactate to creatinine ratio and hospital mortality of pediatric sepsis patients in PICU

Variable	Crude model		Model 1		Model 2		Model 3	
	OR(95% CI)	*P* value	OR(95% CI)	*P* value	OR(95% CI)	*P* value	OR(95% CI)	*P* value
LCR continuous	2.26 (1.97–2.58)	0.000*	2.35 (2.05–2.7)	0.000*	1.25 (1.09–1.44)	0.001*	1.48 (1.25–1.75)	0.000*
LCR, Quartile								
Q1	Ref		Ref	0.000*	Ref		Ref	
Q2	1.23 (0.85–1.78)	0.262	1.28 (0.89–1.86)	0.185	1.15 (0.77–1.72)	0.502	1.41 (0.92–2.16)	0.117
Q3	1.76 (1.24–2.48)	0.001*	1.80 (1.28–2.55)	0.001*	1.49 (1.02–2.19)	0.041*	2.00 (1.32–3.04)	0.001*
Q4	4.36 (3.19–5.94)	0.000*	4.65 (3.39–6.37)	0.000*	1.88 (1.31–2.71)	0.001*	2.94 (1.94–4.45)	0.000*
*P* for trend test		0.000*		0.000*		0.000*		0.000*

Crude model had no adjusted covariates.

Model 1 adjusted for age, sex.

Model 2 adjusted for model 1 plus basic disease, respiratory failure, MAP, PSS.

Model 3 adjusted for model 2 plus WBC, PCT, CRP, BUN, Bilirubin, MV, RRT, vasopressor usage.

^*^P < 0.0005.

*LCR* lactate to creatinine ratio, *MAP* mean arterial pressure, *PSS* Phoenix Sepsis Score, *WBC* white cell count, *PCT* procalcitonin, *CRP* C-reactive protein, *BUN* blood urea nitrogen, *MV* mechanical ventilation, *RRT* renal replacement therapy.

### Restricted cubic spline curves

The threshold effect analysis revealed a nonlinear pattern in the relationship between LCR and hospital mortality in PICU patients with pediatric sepsis (*P* for nonlinearity = 0.018) (Fig. 2). The key inflection point of LCR was determined to be 0.780 via a two-piecewise linear regression model. As shown in the results, when LCR was less than 0.780, there was a significant increase in risk, as evidenced by the mortality odds ratio (OR) of 5.420 (95% CI 2.226–13.198, *P* < 0.005). On the other hand, the link attenuated considerably when LCR surpassed 0.780 (OR = 1.598, 95% CI 1.084–2.355, *P* = 0.018), indicating that additional elevation of LCR did not significantly increase the risk of mortality (Table 3).

**Fig. 2:**
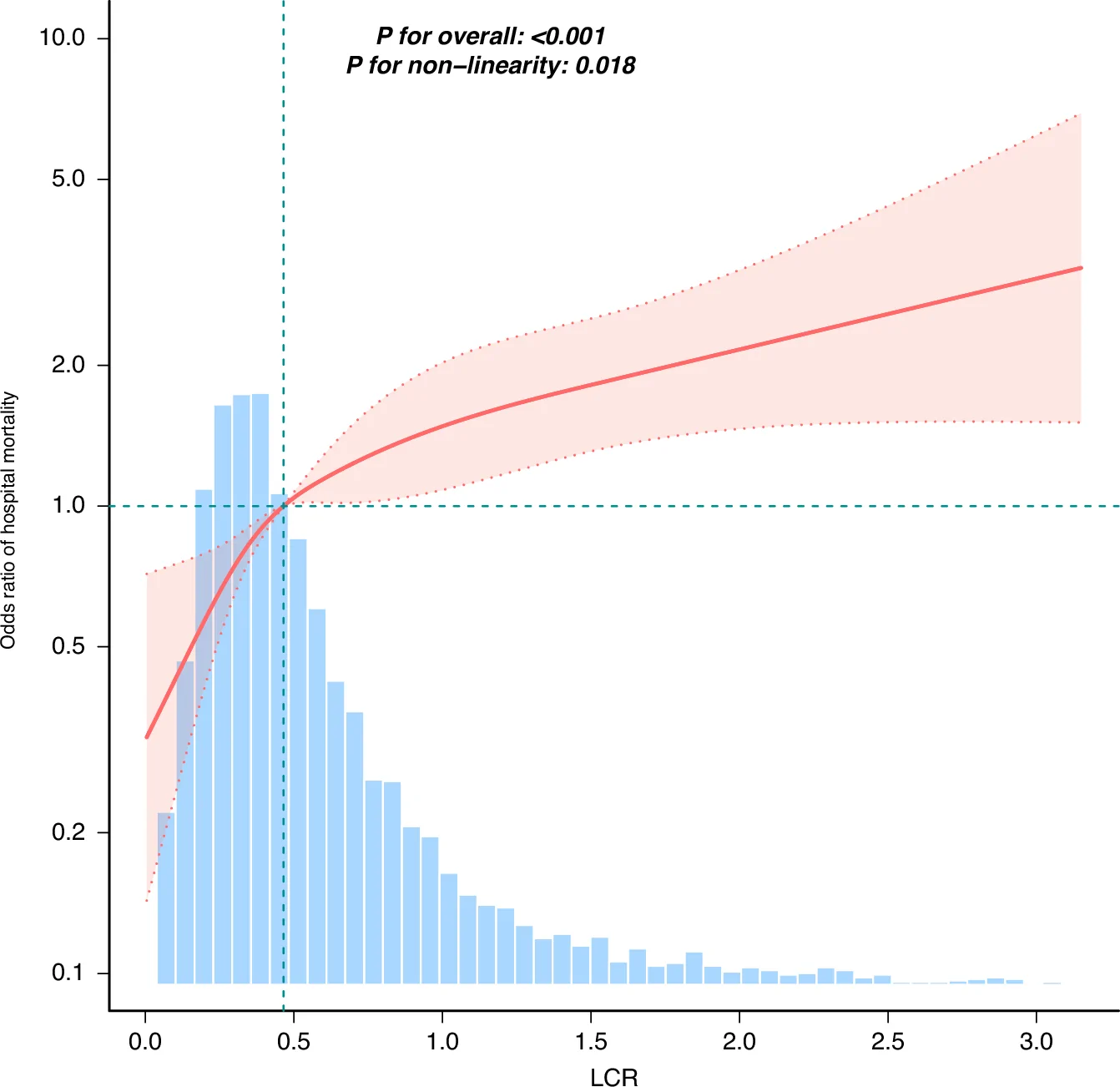
Restricted cubic spline curves of lactate to creatinine ratio (LCR) and hospital mortality in PICU pediatric sepsis patients. Curves show adjusted odds ratios from logistic regression using 99% of the data; adjusted for age, sex, comorbidities, respiratory failure, MAP, PSS, WBC, PCT, CRP, BUN, PF, bilirubin, MV, RRT, and vasopressor use. Shaded areas indicate 95% confidence intervals. LCR lactate to creatinine ratio, MAP mean arterial pressure, PSS Phoenix Sepsis Score, WBC white blood cell count, PCT procalcitonin, CRP C-reactive protein, MV mechanical ventilation, RRT renal replacement therapy.

**Table 3:** Threshold effect analysis of the relationship between lactate to creatinine ratio and hospital mortality of pediatric sepsis patients in PICU

LCR	OR (95%CI)	*P* value
LCR < 0.780	5.420 (2.226–13.198)	0.000*
LCR ≥ 0.780	1.598 (1.084–2.355)	0.018
likelihood ratio test	–	0.019

^*^P < 0.005.

Adjusted factors were age, sex, basic disease, respiratory failure, MAP, PSS, WBC, PCT, CRP, BUN, PF, Bilirubin, MV, RRT, vasopressor usage. Shaded areas around the curves depict a 95% confidence interval.

*OR* Odds Ratio, *CI* confidence interval, *Ref* reference, *LCR* lactate to creatinine ratio, *MAP* mean arterial pressure, *PSS* Phoenix Sepsis Score, *WBC* white cell count, *PCT* procalcitonin, *CRP* C-reactive protein, *MV* mechanical ventilation, *RRT* renal replacement therapy.

### Stratifications and sensitivity analyses

We conducted interactive and stratified analyses to assess the various clinical features. Separate groups and analyses were depending on age (<2 years vs. ≥2 years), sex (male vs. female), basic disease (yes vs. no), vasopressors usage (yes vs. no), and requiring RRT (yes vs. no). The OR of LCR on hospital mortality was consistent across subgroups. Furthermore, no significant interactions were observed within the subgroups except for sex (*P* for interaction > 0.05) (Fig. 3).

**Fig. 3:**
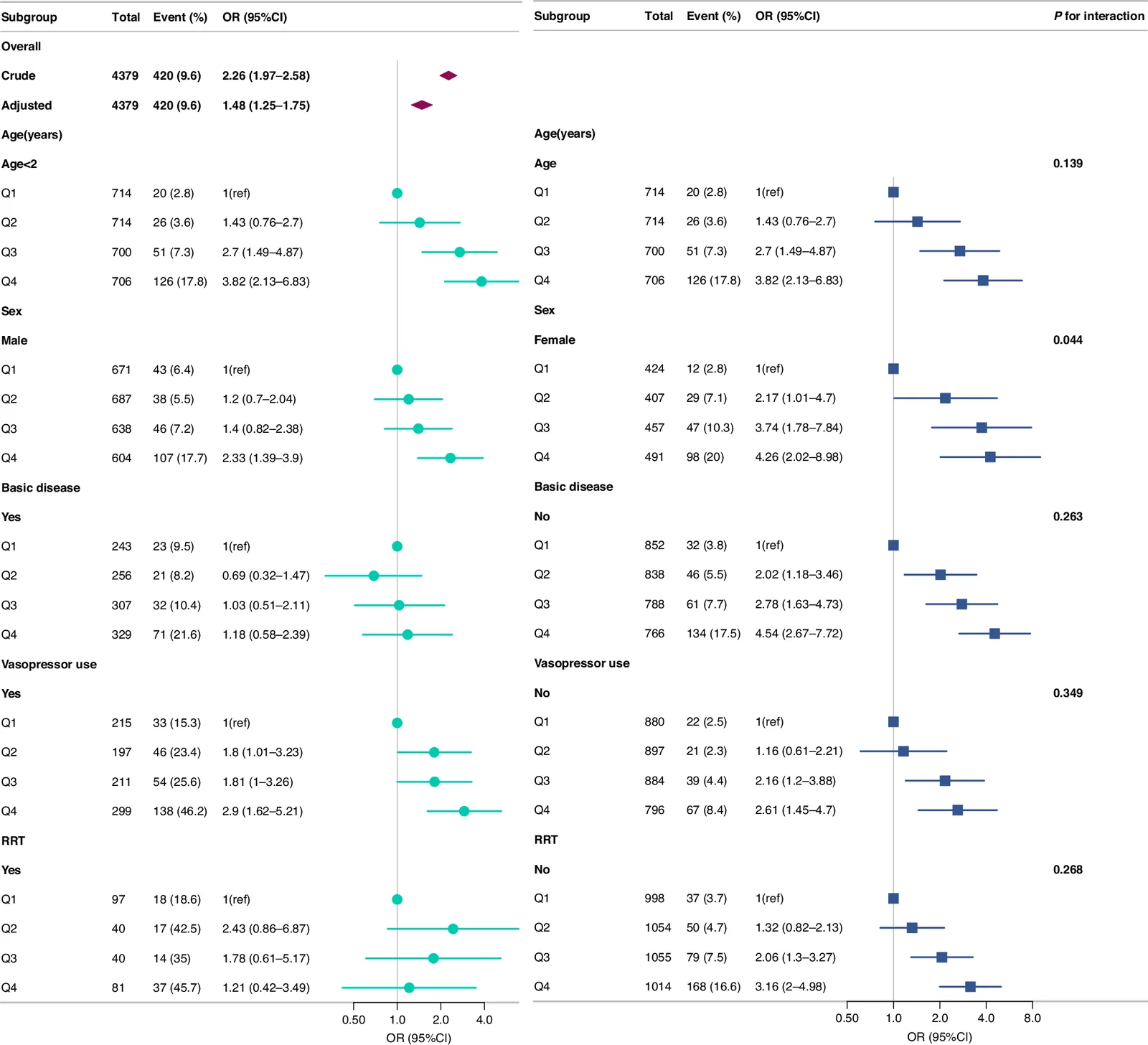
Forest plots of LCR and hospital mortality across different subgroups. Adjusted odds ratios of hospital mortality by lactate to creatinine ratio in different patient subgroups.

Sensitivity analyses in four cohorts (PSS without cardiovascular scores, patients ≥2 years with complete cardiovascular data, excluded outliers and excluding patients on RRT) consistently demonstrated stable associations via multivariate logistic regression (Supplementary Table S2).

## Discussion

This study demonstrated that hospital mortality in pediatric sepsis was positively correlated with LCR even after controlling for potential confounders. To our knowledge, this is the first study to examine the role of serum LCR in pediatric sepsis. Logistic regression analysis indicated that those with the highest LCR had a significantly greater hospital mortality rate than those with lower LCR, and this link remained strong in the subgroup and sensitivity analyses. Restricted cubic spline analysis revealed a nonlinear relationship (*P* for non-linearity = 0.018). When the LCR exceeded 0.780, the risk of hospital mortality did not rise proportionally.

The LCR has been proposed as a biomarker for detecting ischemia-reperfusion injury. Previous research has concentrated mostly on cerebral ischemia and other localized ischemic disorders, notably in the context of newborn asphyxia.^19–21^ Huang et al. demonstrated urinary LCR’s diagnostic utility in hypoxic-ischemic encephalopathy using proton NMR spectroscopy (sensitivity 92%, specificity 88%),^15^ while Thirumurugan et al. validated its role in detecting proximal tubular dysfunction.^22^ Critically, serum LCR surpasses urinary measurements by concurrently reflecting systemic metabolic stress (lactate) and renal functional reserve (creatinine), thereby capturing multidimensional pathophysiological shifts. This translational potential is exemplified in pediatric sepsis. Multivariable analyses revealed LCR’s independent mortality prediction across adjusted models (fully adjusted OR = 1.48, 95% CI:1.25–1.75, *P* < 0.005), with quartile stratification showing a dose-dependent mortality gradient: the highest-risk quartile (Q4) had nearly triple the odds of death (OR = 2.94, 95% CI:1.94–4.45) and a significant trend (*P* < 0.005). The identified critical threshold (LCR < 0.780) marked exponential risk escalation (OR = 5.42), mirroring L-shaped correlations in cardiac arrest-associated AKI,^21^ thus operationalizing LCR as a triage tool. Importantly, LCR retained prognostic value beyond severity scores (PSS), highlighting its unique capacity to decode metabolic-renal dysequilibrium—a hallmark of sepsis pathophysiology. Collectively, these findings position LCR as a pivotal biomarker for risk stratification and therapeutic escalation in pediatric sepsis.

The specific processes tying the LCR to sepsis-related mortality are unknown, but there may be a relationship with acidosis and microcirculatory dysfunction. Circulatory abnormalities in sepsis can cause decreased microcirculatory oxygenation, leading to anaerobic glycolysis and lactate buildup, exacerbating acidosis and impairing microcirculation.^10,23–25^ This, in turn, can impede renal function, resulting in toxin buildup and potentially deadly organ failure.^26–28^ Our findings indicate a link between pediatric sepsis mortality and several clinical parameters, including MAP, WBC, PCT, CRP, lactate, creatinine, BUN, bilirubin, MV, RRT, and vasopressor use. These findings highlight the value of the LCR as a reliable risk stratification indicator in real-world sepsis care. This study supports the use ofthe LCR to determine sepsis severity and guide clinical treatments.

We employed restricted cubic spline curves and a two-piece linear regression model to investigate a possible link between a high LCR and increased hospital mortality in pediatric sepsis. A strong positive correlation was found between the LCR and hospital mortality in pediatric sepsis when the baseline LCR was <0.780, indicating a key threshold below which hospital mortality risk significantly rises. Elevated lactate levels in sepsis suggest insufficient tissue perfusion, and exacerbating factors include underlying injury, a systemic inflammatory response, and microcirculation dysfunction.^29,30^ Similarly, elevated blood creatinine levels indicate the extent of damage to the body caused by an inflammatory response as well as the severity of serious illness. The 2016 International Guidelines for the Management of Sepsis and Septic Shock advocate using serum creatinine as a component of the severity grading system since it can directly measure systemic and microcirculatory perfusion.^31^ Therefore, the LCR threshold of <0.780 may represent an important target for early intervention, aiding in risk stratification and clinical decision-making for pediatric sepsis. Patients with an LCR below this threshold might benefit from more aggressive interventions aimed at enhancing tissue perfusion and renal function, which are crucial for improving sepsis outcomes. Specifically, our findings imply that patients with low LCR may require intensified fluid resuscitation, timely initiation of RRT, and careful adjustment of vasoactive medications to optimize tissue perfusion. For example, an increasing LCR post-resuscitation may indicate better tissue perfusion, while a consistently low LCR may signal the need for additional interventions. Incorporating LCR-guided management into clinical practice could facilitate earlier and more targeted interventions, potentially leading to improved patient outcomes.

Furthermore, we conducted interactive and stratified analyses to assess several clinical features. We looked at subgroups based on age, sex, basic disease, vasopressor usage, and RRT. The consistency of the connection between the LCR and hospital mortality was demonstrated across all subgroups by similarity in multiple tests and the direction of the association. Additionally, we conducted a sensitivity analysis that patients who underwent a PSS assessment without a cardiovascular score, and excluded outliers. The findings were solid, showing a continuous link between LCR and hospital mortality in septic children with varying clinical features.

LCR offers clinical advantages over traditional markers, either lactate or creatinine alone. Creatinine measurements may overlook quick changes in perfusion and metabolic stress, whereas lactate levels represent tissue hypoxia but may not account for renal function. The LCR offers a more comprehensive evaluation of both metabolic and hemodynamic status by combining the two markers. Its threshold effect and nonlinear relationship enable precise risk stratification, helping clinicians identify high-risk patients early and before organ support is required.

This early-warning property distinguishes LCR from therapeutic variables. Mechanical ventilation, vasopressor use, and RRT all showed higher mortality odds in univariate analysis, yet they are typically markers of established organ failure rather than prognostic indicators available at admission. LCR, in contrast, is calculable from routine laboratories upon PICU entry, offering an objective biomarker that is independent of subsequent interventions. Sensitivity analyses excluding patients who received RRT confirmed that LCR retains prognostic value even in the absence of major therapeutic measures.

Despite these advantages, our study has several limitations that should be acknowledged. Firstly, routine laboratory testing was performed only on the first day of PICU admission, limiting continuous monitoring. The lack of repeated measurements prevented evaluation of the impact of lactate and creatinine clearance on patient outcomes. Secondly, the absence of longitudinal LCR measurements beyond the first day precludes analysis of how LCR trends during early resuscitation (e.g., days 1–2) correlate with outcomes, which is particularly relevant given creatinine’s delayed reflection of renal injury and lactate’s rapid fluctuations in response to therapeutic interventions. Baseline data on demographics, vital signs, and underlying diseases were collected to account for potential confounders. Age-specific MAP reference values are published for three pediatric age bands under 2 years (4 weeks–1 month, 1–11 months, and 1–2 years); however, the dataset did not provide this stratification, preventing calculation of MAP-based cardiovascular subscores for these patients. Accordingly, cardiovascular dysfunction was defined using surrogate indicators—serum lactate ≥2 mmol/L or the use of vasoactive agents. Persistent elevation of lactate and the initiation of vasoactive therapy are recommended markers of cardiovascular compromise in pediatric sepsis according to the 2020 Surviving Sepsis Campaign.^17^ Although using these surrogate indicators may introduce misclassification and limit internal and external validity, sensitivity analyses restricted to patients with complete cardiovascular data showed consistent results, supporting the robustness of our findings. This approach aligns with internationally endorsed criteria and ensures methodological consistency across pediatric sepsis studies. In addition, about 8% of patients received RRT, and clearance of creatinine and lactate during therapy may have influenced LCR values. The attenuation of significance after adjustment for RRT suggests that LCR should be interpreted cautiously in this subgroup, as its predictive value may be limited due to altered clearance of lactate and creatinine during RRT. Finally, the generalizability of our findings may be restricted by the use of electronic health records from a single center. Further multicenter studies are needed to validate these results in more diverse populations.

According to our research, there may be a correlation between the LCR and hospital mortality in pediatric sepsis, as higher LCR may be linked to increased hospital mortality. Furthermore, a non-linear relationship was found between LCR and hospital mortality in pediatric sepsis, with increased risk at higher LCR values and a cutoff value of 0.780. These findings indicate the possible involvement of LCR as a risk factor for hospital mortality in pediatric sepsis, implying that LCR may be an effective biomarker.

## Supplementary information

Supplementary materials

## Data Availability

The datasets generated and/or analyzed during the current study are available in the PLOS ONE https://doi.org/10.1371/journal.pone.0306172.
