# Supplementary material for: Association between the lactate to creatinine ratio and hospital mortality in patients with pediatric sepsis: a cohort study
Source: Pediatr Res. 2026 Jan 21;100(2):743–50. doi: 10.1038/s41390-025-04751-1 (PMC13638473; doi:10.1038/s41390-025-04751-1)
Supplement: Supplementary file 1 [file 41390_2025_4751_MOESM1_ESM.pdf]

Supplementary Table S1 Univariate logistic regression analysis of hospital mortality in pediatric sepsis.

Variable	OR (95%CI)	*P* value
Age group		
2 to 5 years	1.44 (1.09–1.92)	0.011
5 to 12 years	1.76 (1.35–2.29)	0.000*
12 to 18 years	2.31 (1.61–3.3)	0.000*
Gender(Male vs Female)	0.85 (0.69–1.04)	0.109
RR(bpm)	0.98 (0.97–0.99)	0.000*
HR(bpm)	1.02 (1.01–1.02)	0.000*
Temperature (°C)	1.25 (1.13–1.38)	0.000*
MAP(mmHg)	0.98 (0.97–0.99)	0.000*
GCS	0.72 (0.7–0.74)	0.000*
PSS	3.26 (2.96–3.58)	0.000*
PCO_2(mmHg)	1.03 (1.02–1.04)	0.000*
PO_2/FiO_2	1 (1–1)	0.000*
WBC ($\times 10^9$/L)	1.02 (1.01–1.02)	0.000*
PLT ($\times 10^9$/L)	1 (1–1)	0.000*
PCT(ng/L)	1.01 (1.01–1.02)	0.000*
CRP(mg/L)	1.01 (1.01–1.01)	0.000*
Lactate(mg/dl)	1.34 (1.3–1.38)	0.000*
Creatinine(mg/dl)	1 (1–1)	0.000*
Bilirubin (mg/dl)	1.01 (1–1.01)	0.000*
BUN(umol/L)	1.06 (1.05–1.08)	0.000*
Fibrinogen (mg/dl)	1.02 (0.99–1.05)	0.255
Basic disease(Yes vs. No)	1.62 (1.31–2)	0.000*
Respiratory failure (Yes vs. No)	2.38 (1.54–3.67)	0.000*
MV (Yes vs. No)	18.23 (5.83–56.98)	0.000*
Vasopressor usage (Yes vs. No)	9.24 (7.44–11.48)	0.000*
RRT (Yes vs. No)	5.67 (4.28–7.52)	0.000*
Hospital length of stay	0.95 (0.94–0.96)	0.000*
PICU length of stay	0.99 (0.98–1)	0.033
LCR	2.26 (1.97–2.58)	0.000*

*P<0.005

LCR, lactate to creatinine ratio; RR, respiratory rate; HR, heart rate; MAP, mean arterial pressure; GCS, glasgow coma score;PSS, Phoenix Sepsis Score; PCO_2, partial pressure of carbon dioxide; PO_2, partial pressure of oxygen; FiO_2, fractional inspired oxygen; PLT, platelet count; WBC, white cell count; PCT, procalcitonin; CRP, C-reactive protein; BUN, blood urea nitrogen; MV, mechanical ventilation; ECMO, extracorporeal membrane oxygenation; RRT, renal replacement therapy.

Supplementary Table S2 Sensitivity analyses.

Variable	Total	Crude model		Model 1		Model 2		Model 3	
		OR(95% CI)	*P* value	OR(95% CI)	*P* value	OR(95% CI)	*P* value	OR(95% CI)	*P* value
**Patients who underwent a PSS assessment without a cardiovascular score**									
LCR continuous	4265	2.40 (2.09 – 2.75)	0.000*	2.52 (2.19 – 2.9)	0.000*	2.10 (1.81 – 2.44)	0.000*	2.09 (1.76 – 2.48)	0.000*
LCR，Quartile									
Q1	1066	Ref		Ref	0.000*	Ref		Ref	
Q2	1066	1.15 (0.8 – 1.68)	0.449	1.20 (0.83 – 1.75)	0.338	1.10 (0.74 – 1.63)	0.635	1.43 (0.92 – 2.22)	0.109
Q3	1066	1.70 (1.2 – 2.4)	0.003*	1.76 (1.24 – 2.5)	0.002*	1.66 (1.14 – 2.4)	0.008*	2.38 (1.56 – 3.64)	0.000*
Q4	1067	4.35 (3.18 – 5.93)	0.000*	4.67 (3.4 – 6.41)	0.000*	3.70 (2.64 – 5.19)	0.000*	4.78 (3.19 – 7.18)	0.000*
P for trend			0.000*		0.000*		0.000*		0.000*
**Patients ⩾ 2 years with complete Phoenix cardiovascular data**									
LCR continuous	1545	2.22 (1.74 – 2.82)	0.000*	2.34 (1.83 – 2.99)	0.000*	1.26 (0.95 – 1.67)	0.104	1.59 (1.14 – 2.21)	0.006
LCR，Quartile									
Q1	386	Ref		Ref		Ref		Ref	
Q2	386	1.19 (0.74 – 1.92)	0.469	1.27 (0.78 – 2.04)	0.335	1.02 (0.58 – 1.78)	0.942	1.30 (0.71 – 2.39)	0.391
Q3	386	1.26 (0.79 – 2.01)	0.340	1.32 (0.82 – 2.12)	0.257	1.00 (0.57 – 1.75)	0.996	1.39 (0.74 – 2.59)	0.304
Q4	387	2.53 (1.65 – 3.88)	0.000*	2.80 (1.80 – 4.33)	0.000*	1.24 (0.73 – 2.11)	0.430	1.93 (1.02 – 3.63)	0.042
P for trend			0.000*		0.000*		0.422		0.042
**Excluded outliers**									
LCR continuous	4335	2.81 (2.42–3.27)	0.000*	2.95 (2.53–3.45)	0.000*	1.42 (1.17–1.71)	0.000*	1.7 (1.38–2.1)	0.000*
LCR，Quartile									
Q1	1083	Ref		Ref		Ref		Ref	
Q2	1083	1.28 (0.89–1.86)	0.188	1.32 (0.91–1.92)	0.141	1.2 (0.8–1.8)	0.379	1.47 (0.96–2.26)	0.076
Q3	1085	1.83 (1.29–2.59)	0.001*	1.86 (1.31–2.64)	0.001*	1.57 (1.06–2.31)	0.023*	2.05 (1.35–3.12)	0.001*
Q4	1083	4.26 (3.11–5.85)	0.000*	4.49 (3.26–6.19)	0.000*	1.91 (1.32–2.76)	0.001*	2.81 (1.85–4.26)	0.000*
P for trend			0.000*		0.000*		0.000*		0.000*
**Excluding patients on RRT**									
LCR continuous	4017	2.4 (2.09-2.75)	0.000*	2.52 (2.19–2.90)	0.000*	2.10 (1.81–2.44)	0.000*	2.09 (1.76–2.48)	0.000*
LCR，Quartile									
Q1	1003	Ref		Ref		Ref		Ref	
Q2	1004	1.15 (0.80–1.68)	0.449	1.20 (0.83–1.75)	0.338	1.10 (0.74–1.63)	0.635	1.43 (0.92–2.22)	0.109
Q3	1005	1.70 (1.20–2.40)	0.003*	1.76 (1.24–2.50)	0.002*	1.66 (1.14–2.40)	0.008	2.38 (1.56–3.64)	0.000*

Q4	1005	4.35 (3.18–5.93)	0.000*	4.67 (3.40–6.41)	0.000*	3.70 (2.64–5.19)	0.000*	4.78 (3.19–7.18)	0.000*
P for trend			0.000*		0.000*		0.000*		0.000*

*$P<0.005$

Crude model had no adjusted covariates. Model 1 adjusted for age, sex.

Model 2 adjusted for model 1 plus basic disease, respiratory failure, MAP, PSS.

Model 3 adjusted for model 2 plus WBC, PCT, CRP, BUN, bilirubin, MV, RRT, vasopressor usage.

LCR, lactate to creatinine ratio; MAP, mean arterial pressure; PSS, Phoenix Sepsis Score; WBC,White cell count; PCT, procalcitonin; CRP, C－reactive protein;BUN,blood urea nitrogen; MV, mechanical ventilation; RRT, renal replacement therapy.
